# Association between cigarette smoking and mortality in patients with hip fracture: A systematic review and meta-analysis

**DOI:** 10.18332/tid/156030

**Published:** 2022-12-12

**Authors:** Nai Zhang, Yu-Juan Liu, Chuang Yang, Peng Zeng, Tao Gong, Lu Tao, Xin-Ai Li

**Affiliations:** 1 Department of Emergency, Jiangxi Province Hospital of Integrated Chinese and Western Medicine, Nanchang, China; 2 Department of Respiratory Medicine, Jiangxi Province Hospital of Integrated Chinese and Western Medicine, Nanchang, China

**Keywords:** meta-analysis, smoking, mortality, hip fracture

## Abstract

**INTRODUCTION:**

Hip fracture is associated with substantial morbidity and mortality, especially among the elderly. Current evidence on the association between cigarette smoking and mortality in hip-fracture patients is controversial. We performed a systematic review and meta-analysis of studies on this association.

**METHODS:**

The databases Medline/PubMed, Embase, Web of Science, and Cochrane Library were searched for studies that estimated the effect of smoking on the risk of mortality in hip-fracture patients. Pooled analyses were conducted of the associations, expressed in relative risk (RR) and 95% confidence intervals (CIs). Heterogeneity was assessed using the I^2^ statistic. Study quality was assessed by the modified Newcastle-Ottawa Scale (NOS) and publication bias was evaluated by a funnel plot, Begg’s and Egger’s tests. Subgroup analyses were performed by study design, race/ethnicity, age ≥60 years, smoking status, and follow-up period.

**RESULTS:**

A total of six articles involving 3739 hip-fracture patients were included in the meta-analysis. Our results indicate that ever-active smoking was significantly associated with an increased risk of death in hip-fracture patients (pooled RR=1.26; 95% CI: 1.08–1.46). In further subgroup analysis, the risk of death was significantly higher in ever-active smokers than in never smokers in White participants (pooled RR=1.23; 95% CI: 1.05–1.44) and elderly aged ≥60 years (pooled RR=1.19; 95% CI: 1.01–1.40), with no significant association in Asian participants (pooled RR=1.42; 95% CI: 0.95–2.11). Current smokers had more risk of death than never smokers (pooled RR=1.26; 95% CI: 1.08–1.46). The association was significant in follow-up periods of ≤1 year (pooled hazard ratio, HR=1.34; 95% CI: 1.05–1.71), 3 years (pooled HR=1.22; 95% CI: 1.05–1.43), and 5 years (pooled HR=1.26; 95% CI: 1.08–1.46).

**CONCLUSIONS:**

Cigarette smoking is associated with an increased risk of mortality in hip-fracture patients, especially in elderly patients aged ≥60 years, current smokers, and White participants. With the extension of follow-up period, the effect on mortality of smoking is profound and lasting.

## INTRODUCTION

Hip fracture has become a global public health problem associated with substantial mortality and heavy social economic burden. Mortality at 1 month after hip fracture is approximately 10%, and mortality within 1 year after hip fracture can reach 36%, despite aggressive management, including surgery and rehabilitation^1-3^. By the year 2040, the estimated annual healthcare costs will reach US$9.8 billion in the United States and $650 million in Canada^4^. This mortality rate has remained relatively stable over time, in contrast to declining mortality rates associated with other causes, such as acute myocardial infarction, underscoring the need to identify preventable risk factors of mortality following hip fracture. Studies showed smoking was associated with an increased risk of hip fracture^5,6^. Tobacco smoking is a global public health threat, especially among the youth and Asian populations^7^, and causes more than seven million deaths annually worldwide^8^. Furthermore, smoking-related diseases are associated with a huge economic burden to individuals and healthcare systems worldwide^9^. These diseases are estimated to contribute to about 5.7% of the global health expenditure. Smoking increases the incidence and mortality of various diseases, including cardiovascular and lung disorders^10-12^.

Currently, the association between cigarette smoking and hip-fracture mortality remains controversial. There have been conflicting views from different studies. Some researches demonstrated that cigarette smoking increased hip-fracture mortality^13,14^, whereas others showed no positive association between the two factors^15-18^. To our knowledge, no systematic review and meta-analysis has demonstrated the association between cigarette smoking and hip-fracture mortality until now. Here, we performed one to assess the associations between the two factors.

## METHODS

This meta-analysis was performed in accordance with Preferred Reporting Items for Systematic Reviews and Meta-analyses guidelines (PRISMA)^19^. The protocol for this meta-analysis is available in PROSPERO (CRD42022315017). All supporting data are available within the article and the Supplementary file.

### Data sources and literature search

Medline/PubMed, Embase, Web of Science, and Cochrane Controlled Register of Trials databases were searched to identify relevant studies evaluating the association between smoking and mortality of hip fracture published until 20 February 2022. The following MeSH terms were used for the search: 1) ‘hip fractures’ or ‘trochanteric fractures’ or ‘intertrochanteric fractures’ or ‘subtrochanteric fractures’; 2) ‘smoking’ or ‘smoker’ or ‘tobacco’ or ‘cigarette’; and 3) ‘prognosis’ or ‘predictor’ or ‘death’ or ‘mortality’ or ‘survival’. The search strategy is shown in Supplementary file Data 1. The references of the included studies were also screened.

### Study selection and inclusion criteria

Studies were selected based on the following inclusion criteria: 1) articles published in English; 2) studies involving in patients with hip fracture^20^; 3) articles reporting multivariate analysis effect estimates on the association between smoking and hip-fracture mortality. For studies based on the same data sources, the articles with the most complete data were included. Review articles, case reports and editorials were excluded.

### Exposure and outcome definitions

Subjects were grouped into ever-active smokers and never smokers, and the risk of hip-fracture mortality was compared between these two groups. Ever-active smokers included current smokers who were smokers when participating in the study and former smokers who already quit smoking before the study period. We combined current and former smokers into one group in the main analysis because many studies did not separately report the estimates for current and former smokers. Never smokers were defined as those who never smoked before participating in the study. We compared the risks of hip fracture-associated mortality separately between ever-active smokers and those who never smoked. The study outcome was mortality in patients diagnosed with hip fracture.

### Study selection and data extraction

Two researchers independently screened the studies for title and abstract. The full text of relevant articles was retrieved. Study eligibility was assessed by checking the research questions, study design, data analysis, outcome, and data availability. Disagreements were resolved through discussions between the two researchers. Effect estimates of hip-fracture mortality with the corresponding 95% confidence intervals (CIs) were obtained from studies involving ever-active smokers. All the extracted effect estimates were recorded into a standard form. The following information was collected from the selected studies: year, mean age, cohort size, study design, target population, smoking status and definitions, outcome variables, and adjusted covariates.

### Quality assessment

Two researchers independently assessed the quality of relevant studies. The quality of studies was evaluated using the Newcastle-Ottawa Scale (NOS)^21^. This 9-point scale assesses three types of biases: selection of study groups (4 points), comparability of cohorts (2 points), and ascertainment of exposure and outcomes (3 points). Quality was scored as: low (0–3), moderate (4–6), and high (7–9 points). Disagreements in the assessment were resolved by consensus. Quality ratings of the studies are shown in Table 1.

**Table 1:** Characteristics of the studies included in the meta-analysis

*Study Year*	*Age (years) mean ± SD (range)*	*Cohort size*	*Country Follow-up period*	*Study design*	*Target population*	*Smoking status and definitions*	*Relative risk (95% CI)*	*Adjustment for covariates*	*NOS score*
Xing et al.^15^ 2021	79.36 ± 7.21	445 Male 171 Female 274	China 1 year	Retrospective observational cohort study	Acute hip fracture (< 7 days); age ≥65 years; underwent surgery; low-energy trauma	Smoking: not mentioned	Multivariate analysis 1.177 (0.496–2.793)	-	8
Wei-Hsiang et al.^16^ 2021	Survivors 71.70 ± 7.36 Nonsurvivors 72.04 ± 7.16	203 Male 114 Female 89	China 30 days	Prospective cohort study	Age ≥60 years; underwent surgery; no systemic diseases	Smoking: not mentioned	Multivariate analysis 1.09 (0.48–2.48)	Age, gender	7
Vosoughi et al.^14^ 2017	75.7 ± 10.6	724 Male 318 Female 406	Iran 3–12 months	Prospective crosssectional study	Age ≥50 years; fracture surgery	Smoking: not mentioned	Multivariate analysis 3 months 1.76 (1.05–2.96) 1 year 1.46 (0.94–2.25)	-	8
Hung et al.^13^ 2014	79.3 ± 7.5 (60–99)	217 Male 61 Female 156	China 35–57 months	Prospective observational study	Age ≥60 years	Smoking: not mentioned	Multivariate analysis 1.7 (1.0–2.9)	-	7
Frost et al.^17^ 2013	Men 79.9 ± 7.7 Women 81.3 ± 7.9	206 Male 51 Female 155	Australia 1–3 years	Prospective epidemiologic investigation	Age ≥60 years	Current/former smoking:smoking one pack-year (even if the smoker had recently given up smoking)	Multivariate analysis 1.41 (0.9–2.2) former and current smokers	Age, gender	7
Söderqvist et al.^18^ 2009	84 (66–103)	1944 Male 491 Female 1453	Sweden 4–24 months	Prospective cohort study	Age ≥66 years	Current smoking: not mentioned	Multivariate analysis 4 months 1.1 (0.7–1.7) Current smokers 2 years 1.1 (0.9–1.4) Current smokers	4 months: Age, gender, ASA, and SPMSQ 2 years: Age, gender, ASA, SPMSQ, and comorbidities	8

NOS: Newcastle-Ottawa scale. ASA: American Society of Anesthesiologists Classification. SPMSQ: Short Portable Mental Status Questionnaire.

### Statistical analysis

The analysis was stratified by smoking status and hip-fracture mortality, using a fixed-effects model when heterogeneity was low or random-effects model for high heterogeneity. Studies were excluded if the relative risk (RR) could not be calculated because of insufficient data or lack of data on CIs. When crude and adjusted hazard ratios (HRs) were reported, adjusted values were used.

The heterogeneity of the studies was tested using the I^2^ statistic^22^. This statistic describes the variance among studies as a proportion of the total variance. A value of I^2^<25% indicates low heterogeneity, 25–50% moderate, >50% to 75% high, and >75% very high heterogeneity. The associated p-value of the heterogeneity of the studies was also calculated, with a non-significant result indicating absence of heterogeneity. Subgroup analyses by study design, race/ethnicity, age ≥60 years, smoking status, and follow-up period, were performed.

Funnel plots of values of log RR and standard error were created to visually evaluate publication bias, and Egger’s regression test^23^ and Begg’s test^24^ were used to statistically assess publication bias. Statistical analysis was conducted using Stata version 14.0. A p-value of less than 0.05 was considered statistically significant.

## RESULTS

### Search results

A total of 10997 relevant studies were retrieved. After removing duplicates, 10278 studies were screened. A total of 534 studies were excluded as these were meta-analyses, systematic reviews, reviews, case reports, and animal studies. Among 9744 eligible articles, 9636 were excluded after abstract screening. Of the remaining 108 studies, 102 were excluded because of irrelevant content (22), conference abstracts without reporting enough findings (23), lack of valuable data (48), and inappropriate outcomes associating smoking and mortality of hip fracture (9). Six studies^13-18^ were included in the meta-analysis (Supplementary file Data 2).

### Study characteristics

The characteristics of the selected studies are shown in Table 1. A total of 3739 participants were evaluated in these studies. One study was retrospective^15^, and five were prospective^13,14,16-18^. The studies were performed in China (3)^13,15,16^, Iran (1)^14^, Australia (1)^17^, and Sweden (1)^18^, and were published between 2009 and 2021. The sample sizes ranged from 203 to 1944 participants. Five studies targeted populations aged ≥60 years^13,15-18^, and one targeted a population aged ≥50 years^14^. The follow-up period varied from 1 to 57 months. Most studies did not clearly define smoking, and some studies adjusted RRs for covariates.

### Quality assessment

Among the 6 included articles, 3 studies had a NOS score of 7, while the other 3 studies had a NOS score of 8. All studies displayed a NOS score of ≥6 (Table 1).

### Smoking status is associated with hip-fracture mortality

*Primary analysis*

**Figure 1:**
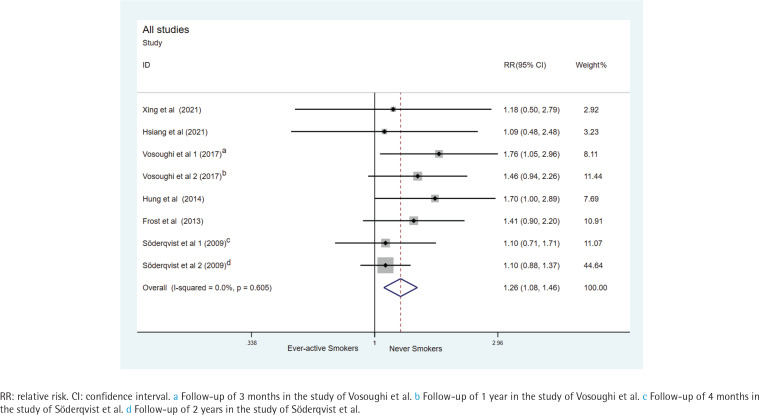
Forest plot of relative risk for hip-fracture mortality between ever-active smokers and never smokers

The RRs for each study and the combined RR for ever-active smokers compared with never smokers are presented in Figure 1. The risk of mortality was significantly higher in ever-active smokers than in never smokers (pooled RR=1.26; 95% CI: 1.08–1.46, Z=3.03, p=0.002), low heterogeneity was found across publications (I^2^=0.0%, p=0.605, fixed-effects model).

*Subgroup analyses*

**Figure 2:**
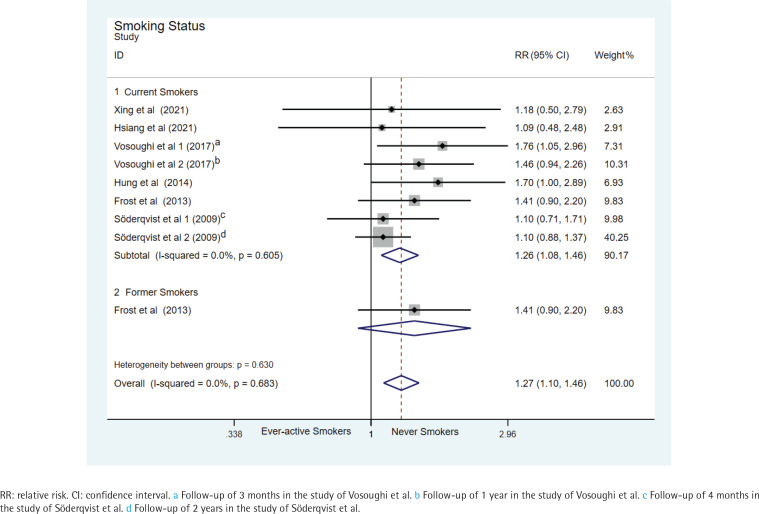
Forest plots of relative risk for hip-fracture mortality between ever-active smokers and never smokers, stratified by smoking status

**Figure 3:**
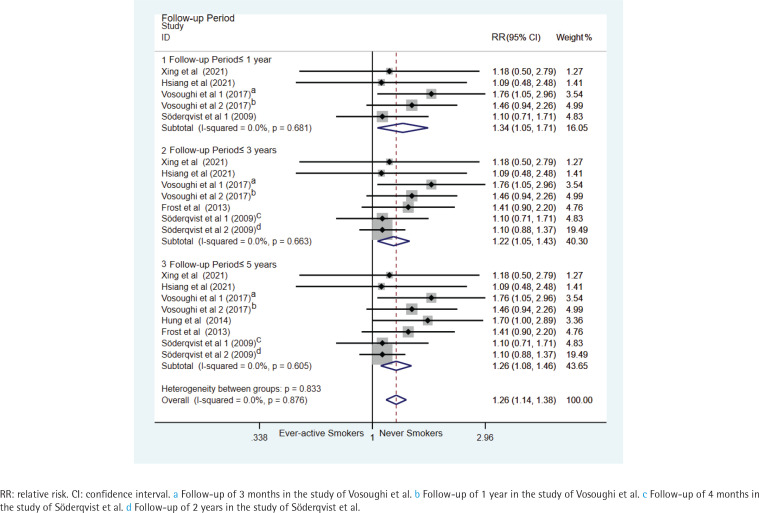
Forest plots of relative risk for hip-fracture mortality between ever-active smokers and never smokers, stratified by follow-up period

Subgroup analysis by study design revealed a significant association between ever-active smoking and an increased risk of hip-fracture mortality in the prospective studies (seven datasets, pooled relative risk RR=1.26, 95% CI: 1.08–1.46) (Supplementary file Data 3). Subgroup analysis by race/ethnicity showed a significant association in White participants (five datasets from three studies, pooled RR=1.23; 95% CI: 1.05–1.44), but no significant association in Asian participants (three studies, pooled RR=1.42; 95% CI: 0.95–2.11) (Supplementary file Data 4). Significant association was also found in subgroup analysis by age (six datasets from five studies, pooled RR=1.19; 95% CI: 1.01–1.40, for age ≥60 years) (Supplementary file Data 5) and current smokers (eight datasets from six studies, pooled RR=1.26; 95% CI: 1.08–1.46) (Figure 2). Furthermore, the association was significant in follow-up periods of ≤1 year (five datasets from four studies, pooled HR=1.34; 95% CI: 1.05–1.71), 3 years (seven datasets from five studies, pooled hazard risk HR=1.22; 95% CI: 1.05–1.43), and 5 years (eight datasets from six studies, pooled HR=1.26; 95% CI: 1.08–1.46), but not significantly linked to a change of mortality in patients with hip fracture with the extension of follow-up period (Figure 3).

*Publication bias*

**Figure 4:**
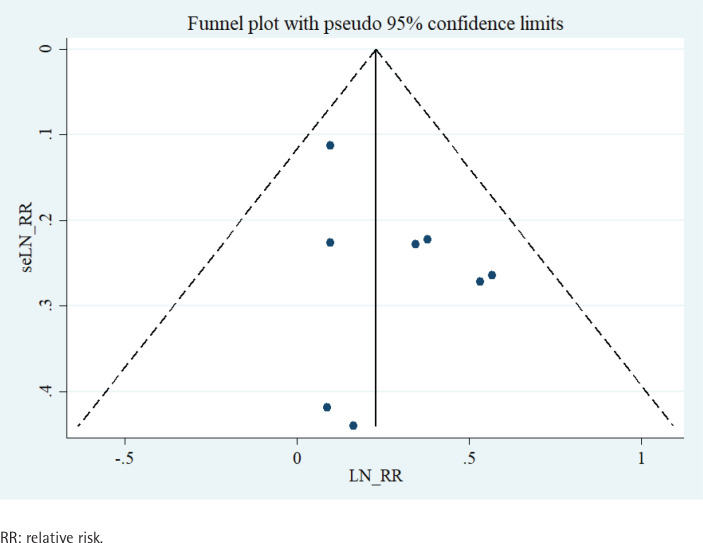
Funnel plot analysis to detect publication bias

Visual evaluation of the funnel plots revealed a symmetrical distribution (Figure 4). Accordingly, Egger’s regression test (p=0.217) and Begg’s test (p=0.536) indicated no statistically significant publication bias among the studies.

## DISCUSSION

Hip fracture is associated with approximately 10% mortality at 1 month and 36% mortality within 1 year, despite aggressive management^1-3^. The overall impact of smoking on the clinical manifestations of hip fracture is unclear. In this study, we presented the findings of our meta-analysis, which was aimed at evaluating the association between smoking and risk of hip-fracture mortality. Our findings indicate that smoking increases the mortality risk from hip fracture, especially in elderly patients aged ≥60 years, current smokers, and White participants. With the extension of follow-up period, the effect on mortality of smoking is profound and lasting. The issue with the association between smoking and hip-fracture mortality suggests the necessity of introducing smoking monitoring to the management of hip fracture and taking effective measures to promote no smoking to reduce mortality in these patients.

The increased risk of hip-fracture mortality in smoking patients can be explained by several mechanisms. Firstly, smoking increases the incidence of various diseases, including malignancy, cardiovascular and pulmonary disorders^10-12,25^. The current study showed that comorbidities significantly increased the risk of mortality after hip fracture^26-28^. Malignancy was reported to be the highest risk factor for mortality in patients who underwent hip-fracture surgery^29^. Cardiovascular disease and pneumonia also increased the risk of death in these patients^26,29^. Individuals with chronic obstructive pulmonary disease (COPD) had a 60–70% higher risk of death following hip-fracture surgery than those without COPD^30^. Therefore, smoking was associated with a higher risk of mortality following hip fracture. Secondly, smoking impairs redox balance, increases the expression of pro-inflammatory cytokines, such as tumor necrosis factor-alpha, interleukin-6, and interleukin-8, and impairs mucociliary clearance and pulmonary immunity, leading to severe infections^31^. This process can cause cytokine release, characterized by the overproduction of inflammatory mediators, which are associated with adverse outcomes in patients with hip fracture^32^. Additionally, cigarette smoking affects soft-tissue structures and peripheral blood flow, increases the risk of infection in operative wounds and soft-tissue flaps, and impairs bone-healing by decreasing osteoblast activity, collagen synthesis, and angiogenesis^33^. These complications may prolong hospital stay and increase mortality.

Hip fracture is very common in elderly patients, especially those aged ≥60^34^. Our study showed ever-active smokers had significantly higher death risk than never smokers in elderly fracture patients. More comorbidities induced by smoking in elderly correlate with higher rates of worse prognosis and mortality, which is consistent with numerous studies^26-28^. Chatterton et al.^28^ reported older age and comorbidity are significantly associated with early in-hospital mortality of hip fracture, with respiratory infections and cardiovascular disease the predominant causes of death. Von et al.^26^ found age at hip fracture was the most important predictor of long-term mortality, where the very elderly only survive for a very short time, with cardiovascular disease as the most common cause of death.

The analyses revealed that current smoking increased the risk of mortality after hip fracture compared with never smoking, with no significant difference between current smokers and former smokers, suggesting that the influence of smoking on hip-fracture mortality would persist even after smoking cessation. It also illustrated that never smoking may be the effective solution to reduce the mortality of hip fracture in comparison to smoking cessation.

Previous studies have shown hip fracture was a significant risk factor contributing the most to long-term as well as short-term excess mortality^1,13,17^. However, few studies assessed the association between smoking and short-term and long-term mortality in hip fracture. Our analysis showed that ever-active smoking was significantly linked to a risk of mortality in hip-fracture patients in follow-up periods of ≤1 year, 3 years and 5 years, indicating the effect of smoking on mortality is profound and lasting with the extension of follow-up period.

### Limitations

This study has some limitations. First, despite that low heterogeneity was found across publications, we cannot rule out the possibility that other inadequately measured factors may bias the associations. Second, although we conducted a comprehensive and systematic literature search with well-defined inclusion and exclusion criteria, most studies did not define smoking status or provided enough data on smoking history (the smoking status questionnaire administered as part of routine care did not assess the duration and amount of smoking by former smokers or smoking cessation during follow-up). Third, although the analysis indicated different outcomes between smoking and hip-fracture mortality in White and Asian participants, more studies with larger sample sizes are needed to confirm the results.

## CONCLUSIONS

This is the first systematic review and meta-analysis on the association between cigarette smoking and mortality in patients with hip fracture. Judging from the present results, the mortality risk was higher in ever-active smokers, especially in elderly patients aged ≥60 years, current smokers and White participants. With the extension of follow-up period, the effect of smoking on mortality is profound and lasting. The high mortality rate in these patients should serve as an incentive, for the patients and also for at-risk populations, to quit smoking. In addition, more data on smoking status should be analyzed to accurately estimate the effects of smoking on hip-fracture mortality.

## Data Availability

The data supporting this research are available from the authors on reasonable request.
