# Supplementary material for: Association between cigarette smoking and mortality in patients with hip fracture: A systematic review and meta-analysis
Source: Tob Induc Dis. 2022 Dec 12;20:110. doi: 10.18332/tid/156030 (PMC9743796; doi:10.18332/tid/156030)
Supplement: Supplementary file 1 [file TID-20-110-s1.pdf]

Data S1.

Detailed Search Strategies

PubMed：

Search: ((("Hip Fractures"[Mesh]) OR (((((((Fractures, Hip[Title/Abstract]) OR (Trochanteric Fractures[Title/Abstract])) OR (Fractures, Trochanteric[Title/Abstract])) OR (Intertrochanteric Fractures[Title/Abstract])) OR (Fractures, Intertrochanteric[Title/Abstract])) OR (Subtrochanteric Fractures[Title/Abstract])) OR (Fractures, Subtrochanteric[Title/Abstract])))

AND

(((("Smoking"[Mesh]) OR ((((((((Smoking Behaviors[Title/Abstract]) OR (Behavior, Smoking[Title/Abstract])) OR (Behaviors, Smoking[Title/Abstract])) OR (Smoking Behavior[Title/Abstract])) OR (Smoking Habit[Title/Abstract])) OR (Habit, Smoking[Title/Abstract])) OR (Habits, Smoking[Title/Abstract])) OR (Smoking Habits[Title/Abstract]))) OR (("Smokers"[Mesh]) OR ((((((((((((Smoker[Title/Abstract]) OR (Smokers, Non-Tobacco Products[Title/Abstract])) OR (Non-Tobacco Products Smoker[Title/Abstract])) OR (Non-Tobacco Products Smokers[Title/Abstract])) OR (Smoker, Non-Tobacco Products[Title/Abstract])) OR (Smokers, Non Tobacco Products[Title/Abstract])) OR (Vapers[Title/Abstract])) OR (Vaper[Title/Abstract])) OR (Smokers, Tobacco[Title/Abstract])) OR (Smoker, Tobacco[Title/Abstract])) OR (Tobacco Smoker[Title/Abstract])) OR (Tobacco Smokers[Title/Abstract])))) OR (("Tobacco Products"[Mesh]) OR (((((((((((((((((((((((((((((Product, Tobacco[Title/Abstract]) OR (Products, Tobacco[Title/Abstract])) OR (Tobacco Product[Title/Abstract])) OR (Cigarillos[Title/Abstract])) OR (Cigarillo[Title/Abstract])) OR (Pipe Tobacco[Title/Abstract])) OR (Pipe Tobaccos[Title/Abstract])) OR (Tobacco, Pipe[Title/Abstract])) OR (Tobaccos, Pipe[Title/Abstract])) OR (Cigars[Title/Abstract])) OR (Cigar[Title/Abstract])) OR (Kreteks[Title/Abstract])) OR (Kretek[Title/Abstract])) OR (Kreteks Tobacco[Title/Abstract])) OR (Kreteks Tobaccos[Title/Abstract])) OR (Tobacco, Kreteks[Title/Abstract])) OR (Tobaccos, Kreteks[Title/Abstract])) OR (Bidis[Title/Abstract])) OR (Bidi[Title/Abstract])) OR (Bidis Tobacco[Title/Abstract])) OR (Bidis Tobaccos[Title/Abstract])) OR (Tobacco, Bidis[Title/Abstract])) OR (Tobaccos, Bidis[Title/Abstract])) OR (Tobacco, Bidi[Title/Abstract])) OR (Bidi Tobacco[Title/Abstract])) OR (Bidi Tobaccos[Title/Abstract])) OR (Tobaccos, Bidi[Title/Abstract])) OR (Cigarettes[Title/Abstract])) OR (Cigarette[Title/Abstract])))))

AND

(prognosis[MeSH:noexp] OR diagnosed[Title/Abstract] OR cohort*[Title/Abstract] OR cohort effect[MeSH Term] OR cohort studies[MeSH:noexp] OR predictor*[Title/Abstract] OR death[Title/Abstract] OR "models, statistical"[MeSH Term])

Limit: English

EMBASE：

'hip fractures' OR 'Fractures,Hip':ab,ti OR 'Trochanteric Fractures':ab,ti OR 'Fractures,Trochanteric':ab,ti OR 'Intertrochanteric Fractures':ab,ti OR 'Fractures, Intertrochanteric':ab,ti OR 'Subtrochanteric Fractures':ab,ti OR 'Fractures,Subtrochanteric':ab,ti

AND

'smoking' OR 'smoking behaviors':ab,ti OR 'behavior, smoking':ab,ti OR 'behaviors, smoking':ab,ti OR 'smoking behavior':ab,ti OR 'smoking habit':ab,ti OR 'habit, smoking':ab,ti OR 'habits, smoking':ab,ti OR 'smoking habits':ab,ti OR 'smokers' OR 'Smoker':ab,ti OR 'Smokers,Non-Tobacco Products':ab,ti OR 'Non-Tobacco Products Smoker':ab,ti OR 'Non-Tobacco Products Smokers':ab,ti OR 'Smoker, Non-Tobacco Products':ab,ti OR 'Smokers, Non Tobacco Products':ab,ti OR 'Vapers':ab,ti OR 'Vaper':ab,ti OR 'Smokers, Tobacco':ab,ti OR 'Smoker, Tobacco':ab,ti OR 'Tobacco Smoker':ab,ti OR 'Tobacco Smokers':ab,ti OR

'product, AND tobacco' OR 'Products,Tobacco':ab,ti OR 'Tobacco Product':ab,ti OR 'Cigarillos':ab,ti OR 'Cigarillo':ab,ti OR 'Pipe Tobacco':ab,ti OR 'Pipe Tobaccos':ab,ti OR 'Tobacco, Pipe':ab,ti OR 'Tobaccos,Pipe':ab,ti OR 'Cigars':ab,ti OR 'Cigar':ab,ti OR 'Kreteks':ab,ti OR 'Kretek':ab,ti 'Kreteks Tobacco':ab,ti OR 'Kreteks Tobaccos':ab,ti OR 'Tobacco, Kreteks':ab,ti OR 'Tobaccos, Kreteks':ab,ti OR 'Bidis':ab,ti OR 'Bidi':ab,ti OR 'Bidis Tobacco':ab,ti OR 'Bidis Tobaccos':ab,ti OR 'Tobacco, Bidis':ab,ti OR 'Tobaccos, Bidis':ab,ti OR 'Tobacco, Bidi':ab,ti OR 'Bidi Tobacco':ab,ti 'Bidi Tobaccos':ab,ti OR 'Tobaccos, Bidi ':ab,ti OR 'Cigarettes':ab,ti OR 'Cigarette':ab,ti

AND

'prognosis':ab,ti OR 'diagnosed':ab,ti OR 'cohort*':ab,ti OR 'cohort effect':ab,ti OR 'cohort studies':ab,ti OR 'predictor*':ab,ti OR 'death':ab,ti OR 'models, statistical':ab,ti

Limit: English

Web of Science:

TS=(hip fractures OR Fractures, Hip OR Trochanteric Fractures OR Fractures, Trochanteric OR Intertrochanteric Fractures OR Fractures, Intertrochanteric OR Subtrochanteric Fractures OR Fractures, Subtrochanteric)

AND

TS=(smoking OR Smoking Behaviors OR Behavior, Smoking OR Behaviors, Smoking OR Smoking Behavior OR Smoking Habit OR Habit, Smoking OR Habits, Smoking OR Smoking Habits OR smokers OR Smoker OR Smokers, Non-Tobacco Products OR Non-Tobacco Products Smoker OR Non-Tobacco Products Smokers OR Smoker, Non-Tobacco Products OR Smokers, Non Tobacco Products OR Vapers OR Vaper OR Smokers, Tobacco OR Smoker, Tobacco OR Tobacco Smoker OR Tobacco Smokers OR Product, Tobacco OR Products, Tobacco OR Tobacco Product OR Cigarillos OR Cigarillo OR Pipe Tobacco OR Pipe Tobaccos OR Tobacco, Pipe OR Tobaccos, Pipe OR Cigars OR Cigar OR Kreteks OR Kretek OR Kreteks Tobacco OR Kreteks Tobaccos OR Tobacco, Kreteks OR Tobaccos, Kreteks OR Bidis OR Bidi OR Bidis Tobacco OR Bidis Tobaccos OR Tobacco, Bidis OR Tobaccos, Bidis OR Tobacco, Bidi OR Bidi Tobacco OR Bidi Tobaccos OR Tobaccos, Bidi OR Cigarettes OR Cigarette)

AND

TS=(prognosis OR diagnosed OR cohort* OR cohort effect OR cohort studies OR predictor* OR death OR models, statistical)

Limit: English

Cochrance Library:

(Fractures, Hip):ab,ti,kw OR (Trochanteric Fractures):ab,ti,kw OR (Fractures, Trochanteric):ab,ti,kw OR (Intertrochanteric Fractures):ab,ti,kw OR (Fractures, Intertrochanteric):ab,ti,kw OR (Subtrochanteric Fractures):ab,ti,kw OR (Fractures, Subtrochanteric):ab,ti,kw

AND

(Smoking):ab,ti,kw OR (Smoking Behaviors):ab,ti,kw OR (Behavior, Smoking):ab,ti,kw OR (Behaviors, Smoking):ab,ti,kw OR (Smoking Behavior):ab,ti,kw OR (Smoking Habit):ab,ti,kw OR (Habit, Smoking):ab,ti,kw OR (Habits, Smoking):ab,ti,kw OR (Smoking Habits):ab,ti,kw OR (Smokers):ab,ti,kw OR (Smoker):ab,ti,kw OR (Smokers, Non-Tobacco Products):ab,ti,kw OR

(Non-Tobacco Products Smoker):ab,ti,kw OR (Non-Tobacco Products Smokers):ab,ti,kw OR (Smoker, Non-Tobacco Products):ab,ti,kw OR (Smokers, Non Tobacco Products):ab,ti,kw OR (Vapers):ab,ti,kw OR (Vaper):ab,ti,kw (Smokers, Tobacco):ab,ti,kw OR (Smoker, Tobacco):ab,ti,kw OR (Tobacco Smoker):ab,ti,kw OR (Tobacco Smokers):ab,ti,kw OR (Product, Tobacco):ab,ti,kw OR (Products, Tobacco):ab,ti,kw OR (Tobacco Product):ab,ti,kw OR (Cigarillos):ab,ti,kw OR (Cigarillo):ab,ti,kw OR (Pipe Tobacco):ab,ti,kw OR (Pipe Tobaccos):ab,ti,kw OR (Tobacco, Pipe):ab,ti,kw OR (Tobaccos, Pipe):ab,ti,kw (Cigars):ab,ti,kw OR (Cigar):ab,ti,kw OR (Kreteks):ab,ti,kw OR (Kretek):ab,ti,kw OR (Kreteks Tobacco):ab,ti,kw OR (Kreteks Tobaccos):ab,ti,kw OR (Tobacco, Kreteks):ab,ti,kw OR (Tobaccos, Kreteks):ab,ti,kw OR (Bidis):ab,ti,kw OR (Bidi):ab,ti,kw OR (Bidis Tobacco):ab,ti,kw OR (Bidis Tobaccos):ab,ti,kw (Tobacco, Bidis):ab,ti,kw OR (Tobaccos, Bidis):ab,ti,kw OR (Tobacco, Bidi):ab,ti,kw OR (Bidi Tobacco):ab,ti,kw OR (Bidi Tobaccos):ab,ti,kw OR (Tobaccos, Bidi):ab,ti,kw OR (Cigarettes):ab,ti,kw OR (Cigarette):ab,ti,kw

AND

(prognosis):ab,ti,kw OR (diagnosed):ab,ti,kw OR (cohort*):ab,ti,kw OR (cohort effect):ab,ti,kw OR (cohort studies):ab,ti,kw OR (predictor*):ab,ti,kw OR (death):ab,ti,kw OR (models, statistical):ab,ti,kw

Limit: English

Data S2.

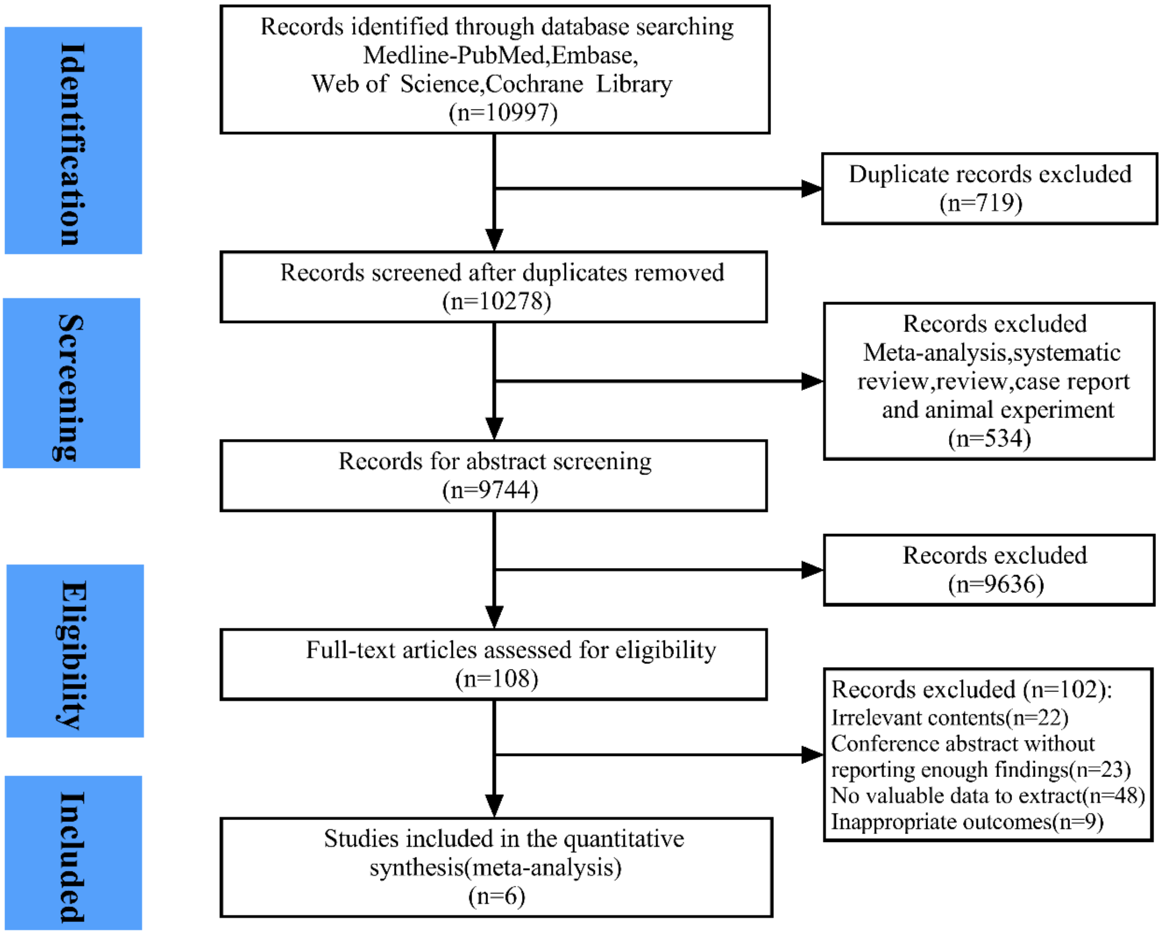

Figure Flowchart of study selection.

Data S3.

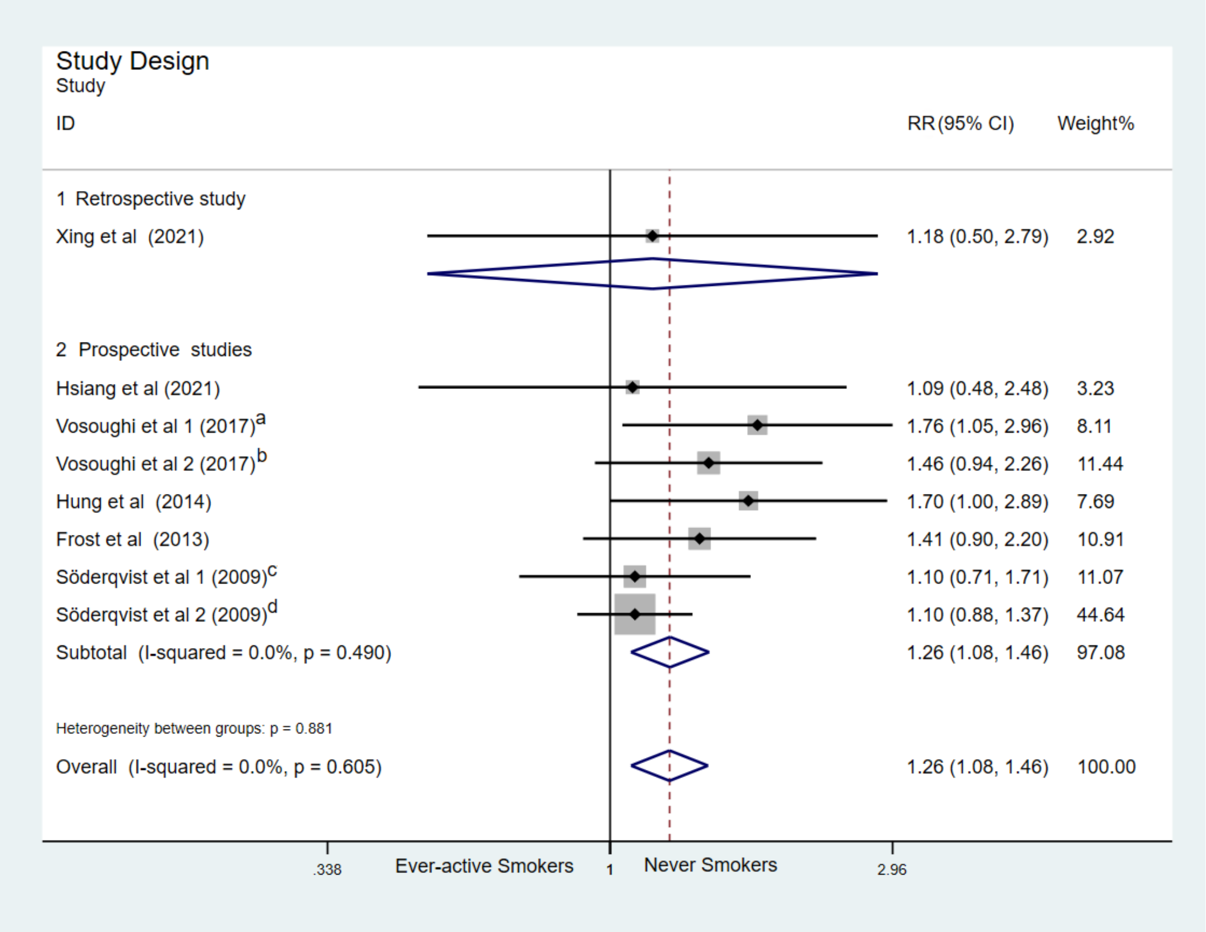

Figure Forest plots of relative risk for hip fracture mortality between ever-active smokers and never smokers stratified by study design.

a, follow-up of 3 months in the study of Vosoughi et al.

b, follow-up of 1 year in the study of Vosoughi et al.

c, follow-up of 4 months in the study of Söderqvist et al.

d, follow-up of 2 years in the study of Söderqvist et al.

RR, relative risk.

CI, confidence interval.

Data S4.

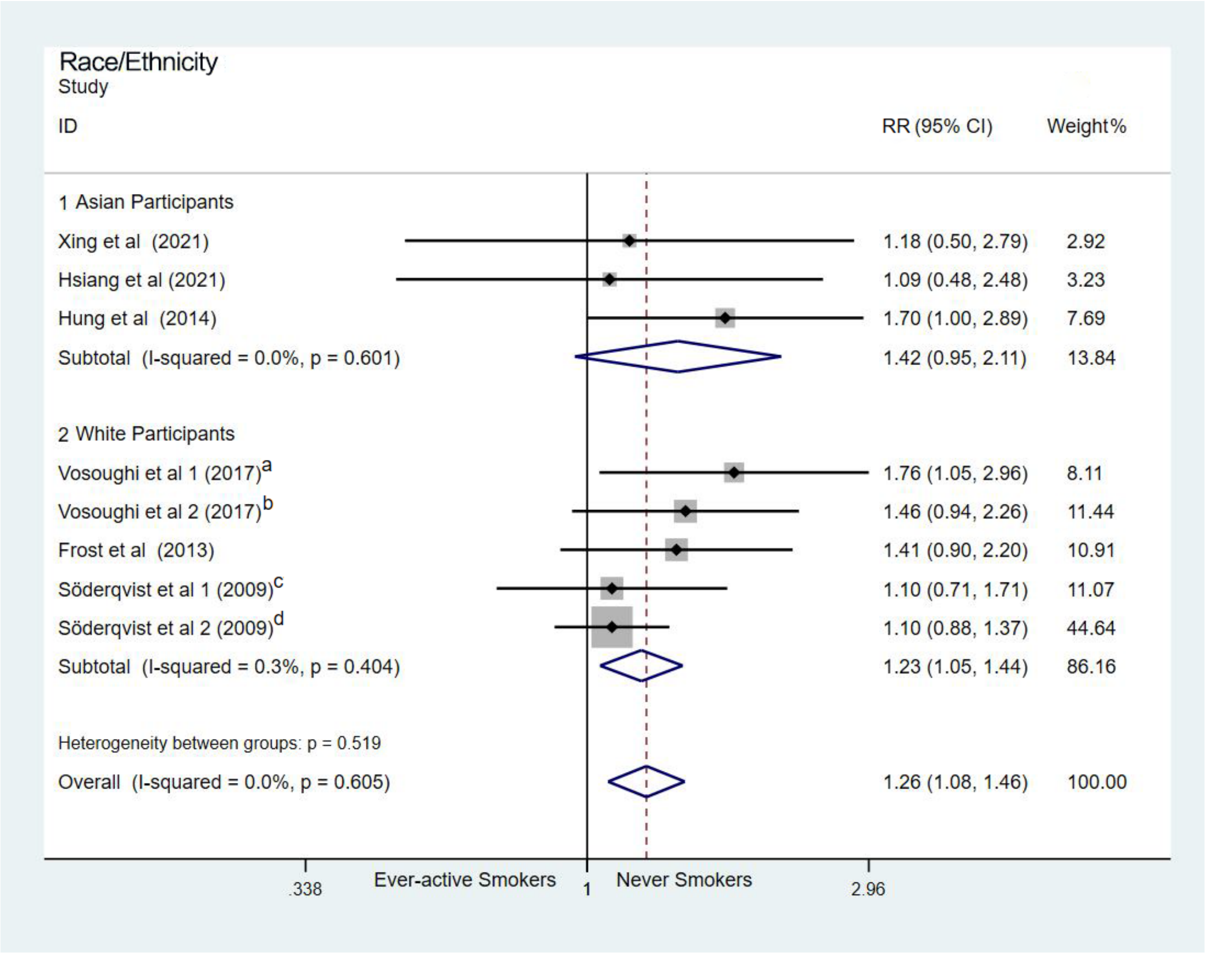

Figure Forest plots of relative risk for hip fracture mortality between ever-active smokers and never smokers stratified by Race/Ethnicity.

a, follow-up of 3 months in the study of Vosoughi et al.

b, follow-up of 1 year in the study of Vosoughi et al.

c, follow-up of 4 months in the study of Söderqvist et al.

d, follow-up of 2 years in the study of Söderqvist et al.

RR, relative risk.

CI, confidence interval.

Data S5.

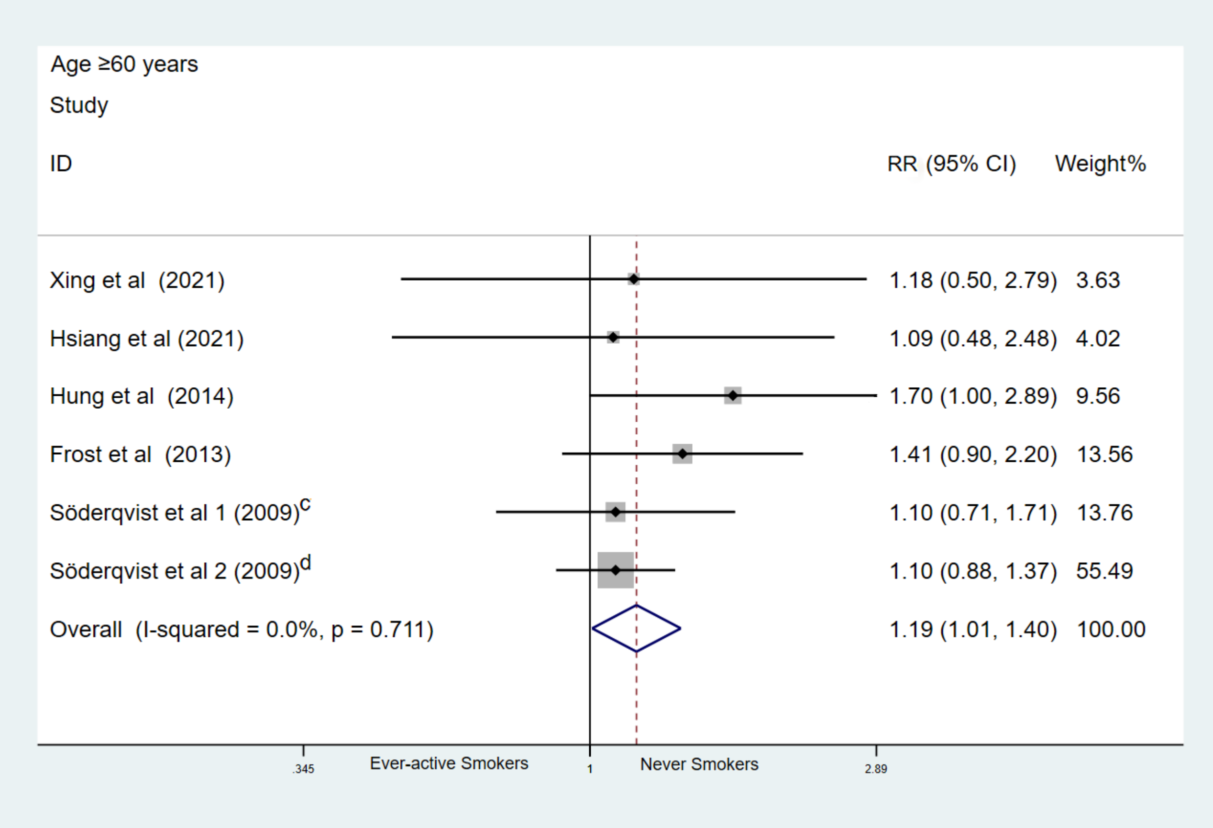

Figure Forest plots of relative risk for hip fracture mortality between ever-active smokers and never smokers stratified by Age ≥60 years.

c, follow-up of 4 months in the study of Söderqvist et al.

d, follow-up of 2 years in the study of Söderqvist et al.

RR, relative risk.

CI, confidence interval.

© 2022 Zhang N. et al.
